# Molecular Sex Identification in Dioecious *Hippophae rhamnoides* L. via RAPD and SCAR Markers

**DOI:** 10.3390/molecules23051048

**Published:** 2018-05-01

**Authors:** Wu Zhou, Yuwei Wang, Gong Zhang, Guangxiang Luan, Shasha Chen, Jing Meng, Honglun Wang, Na Hu, Yourui Suo

**Affiliations:** 1 Qinghai Key Laboratory of Qinghai-Tibet Plateau Biological Resources, Northwest Institute of Plateau Biology, Chinese Academy of Sciences, Xining 810008, China; zhouwu870624@163.com (W.Z.); wangyuwei12@mails.ucas.ac.cn (Y.W.); qinghaizhanggong@126.com (G.Z.); luanguangxiang1990@126.com (G.L.); chenshasha15@mails.ucas.ac.cn (S.C.); greensakural@hotmail.com (J.M.); hlwang@nwipb.cas.cn (H.W.); 2 State Key Laboratory of Plateau Ecology and Agriculture, Qinghai University, Xining 810016, China; 3 University of the Chinese Academy of Sciences, Beijing 100049, China

**Keywords:** *Hippophae rhamnoides* L, dioecious, sex determination, RAPD, SCAR

## Abstract

The dioecious property of the sea buckthorn (*Hippophae rhamnoides* L.) prevents sex recognition via traditional observation at the juvenile stage, thus impeding breeding and economic cropping; A random amplified polymorphic DNA (RAPD) and a sequence characterized amplified region (SCAR) markers were used to identify the sexes. A total of 45 random decamer primers were used to screen genomic DNA pools of staminate and pistillate genotypes for genetic polymorphisms. One female sex-linked marker was identified. D15 (5′-CATCCGTGCT-3′) amplified a particular band of 885 bp, which showed polymorphism among staminate and pistillate genotype plants. The SCAR marker Hrcx-15 was obtained by sequencing the fragment. The alleles of 140 pistillate genotypes were examined but not of the 140 staminate genotypes discerned via taxonomy. Staminate and pistillate genotypes of sea buckthorn plants can be distinguished, using Hrcx-15 as a genetic marker for sex identification and for expediting cultivation for commercial applications.

## 1. Introduction

*Hippophae rhamnoides* L. (sea buckthorn), is a multipurpose forest tree that belongs to the family *Elaeagnaceae* of the genus *Hippophae* L., which encompasses seven species and twelve subspecies that are widely distributed throughout the world [1,2]. Natural *H. rhamnoides* is widespread throughout the temperate regions of Asia and Europe, with a distribution in the Himalayan Mountains at high altitude, and usually reproducing on sandy riverbanks or coastal dunes. Almost 90% of the world resources of *H. rhamnoides* grow in China and the total area may approasch or exceed 1.5 million hectares. *H. rhamnoides* L. subsp. Sinensis Rousi, a main subspecies and a primitive member of the genus, is endemic to China. This subspecies ranges from the Northeastern part of Tibet to the southwestern corner of the Da Hinggan Mountains, through the Loess Plateau.

Only about 6% of all 240,000 angiosperm species (14,620 of 240,000) are dioecious [3]. Gender determination of dioecy is generally under the influence of a cytogenetically identified sex chromosome system. Different systems of sex determination via sex chromosomes have been reported for several dioecious plants [4,5,6]. XY sex chromosome systems feature male heterogamety (homogametic sex in ZZ systems) [7,8,9,10], while females are homogametic XX (heterogametic sex in ZW systems) [11,12,13,14]. Some other species (e.g., *Rumex acetosa*) feature an XY “dosage system” of sex determination, in which the sex of an individual is determined by the ratio of the number of X chromosomes to the number of sets of autosomes (X/A ratio) [15]. In addition, the gender of some dioecious species was influenced by the genetic segregation of alleles at one or several loci on autosomes [16,17], since not all dioecious plants are bound to have morphologically separate sex chromosomes [18]. For some dioecious species, environmental factors have been described to be involved in the determination of sex or the modulation of sex expression. [19]. *Hippophae rhamnoides* L. (sea buckthorn) has a polymorphic shrubby diploid genotype with a basic composition of 2n = 24 chromosomes. Among these, 11 pairs consist of autosomes and the one remaining pair consists of sex-chromosomes [20]. In the plant kingdom, many molecular mechanisms modulate the sex determination and many studies investigated the gender determination of dioecy. However, our knowledge of the genetic basis for sex determination of dioecious species is rather limited: no definite theories exist about genetic mechanisms that control sexual dimorphism in plants. In some plants, sex is determined by a simple mendelian genetic system based on the segregation of one locus, as in the squirting cucumber (*Ecballium elaterium*), or of a few loci, as in the annual mercury (*Mercurialis annua*).

Although the chromosome number and the cytogenetically identified sex chromosome in *Hippophae rhamnoides* L. has been established, which make it possible to sex them by karyotype. The common practice of identifying sex chromosomes by genetic mapping is also difficult to begin with. DNA or RNA-based molecular markers can be used to identify sex in dioecy at the molecular level. Sex-linked markers amplified by Polymerase Chain Reaction (PCR) have been reported in several plant species, including some crops and economically valuable plants [21,22,23]. Most of these studies identified sex-specific bands, which are amplified by Random Amplified Polymorphic DNA (RAPD) and have been cloned and sequenced. Based on the sequences of the fragments, primers specifically amplifying the bands have been designed. In general, these Sequence-Characterized Amplified Regions (SCARs) are more stably amplified than RAPD, as they can be amplified at higher annealing temperatures and can identify the sex of juvenile individuals of commercially valuable plants. 

The berries of *H. rhamnoides* have a strong acidic taste, and the nutritious juice is rich in vitamins (A, C, E, K, and P). In addition, clinical studies have shown that the berries boost the immune system, possess potential antitumor activity, enhance radiation protection, and improve skin problems [24,25,26,27,28]. However, as with the majority of dioecious perennials, a unique feature of *H. rhamnoides* is that neither male nor female trees can be differentiated until the berries appear, which requires 5–7 years after seed germination [29]. Economic benefits can be increased if low-value-added male seedlings can be abandoned at the juvenile stage via apparent markers. Consequently, pistillate trees should account for the majority and an optimal proportion of 6–7% of male genotypes is considerably sufficient for providing sperm [30]. Thus, an effective and easy-to-use method to differentiate staminate from pistillate genotypes at the seedling stage is required to maintain the optimal ratio between male and female plants. Such a method has not been reported to date. Molecular bio-technology allows screening and diagnosing of gene polymorphism markers based on sex-linked DNA sequences, which can be applied long before pheno-morphological features appear. This study implemented staminate- or pistillate-specific genetic markers to identify the sex of *H. rhamnoides* during the vegetative growth phase.

## 2. Results

### 2.1. Sex-Linked RAPD Marker

**Figure 1:**
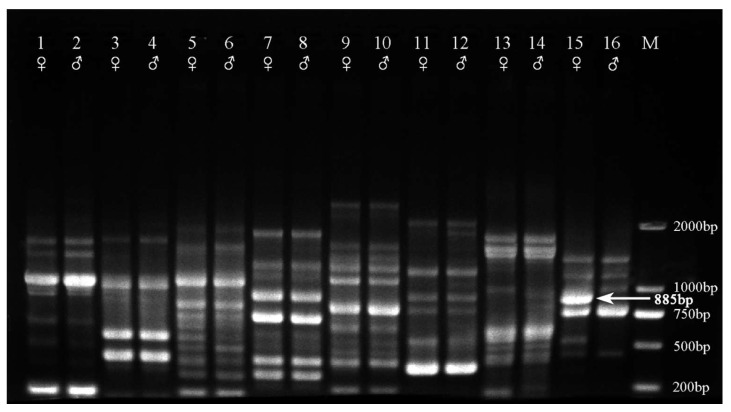
Part of the RAPD screening products of bulk DNA from each of the 140 male and female *H. rhamnoides* samples via decamer primers (Lanes 1, 3, 5, 7, 9, 11, 13, and 15: female; Lanes 2, 4, 6, 8, 10, 12, 14, and 16: male). The female-specific 885 bp band is indicated with an arrow. M, DNA Marker DL2000 (Takara, Dalian, China).

**Figure 2:**
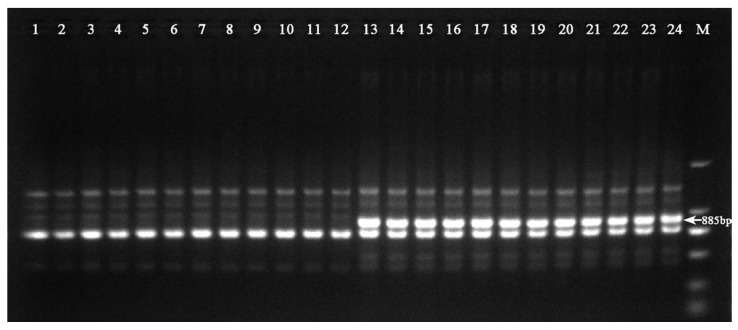
Amplification profile of RAPD primer D15 in *H. rhamnoides* genotypes. The arrow indicates 885 bp, lanes 1–12: male, lanes 13–24: female. M, DNA Marker DL2000 (Takara, Dalian, China).

Genetic polymorphism among two bulks (female and male genotypes) were screened via RAPD random decamer primers. Amplification of polymorphic bands between male and female genotypes were obtained from most RAPD primers. Among all 45 RAPD primers (decamer) screened, only one primer consistently showed a unique 885 bp fragment (D15 (5′-AATCGGGCTG-3′; hereafter named Hrcx-15)) and was particularly pronounced in the female bulk samples (Figure 1). This sex-linked molecular fragment was found to be reproducible. To test both the reliability and stability of the Hrcx-15, RAPD-PCR was repeated three times, using 140 individual staminate and pistillate genomic DNAs as template. A specific band was found in all females, while this was missing in all male samples (Figure 2). 

### 2.2. DNA Sequence of Female-Specific Fragments

**Figure 3:**
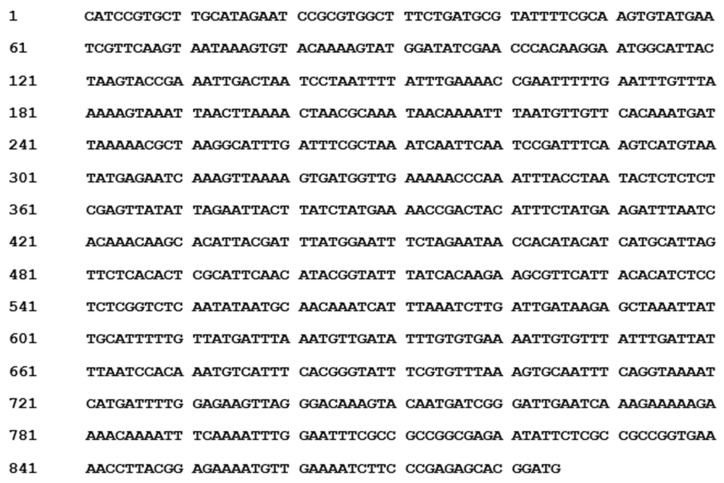
Nucleotide Sequence of the RAPD Amplicon Hrcx-15.

The sequenced nucleotide information (Figure 3) of the 885 bp fragment presented in the female genotypes from a colony of molecular marker Hrcx-15 was submitted to the National Center for Biotechnology Information (NCBI) GenBank. The Basic Local Alignment Search Tool (BLAST) algorithm was performed to analyze DNA and protein sequence similarities with the public database stored at NCBI server. The megaBLAST analysis of the Hrcx-15 sequence showed partial homology with the *Vitis vinifera* genome shotgun sequence AM462442.2. However, a BLASTN search of the Hrcx-15 sequence demonstrated a sequence homology (*E*-value of 0.35, 10% query coverage) with a protein of *Frangula alnus* subsp. alnus, and a similarity with a protein of *Cyprinus carpio* (*E*-value of 6.1, 13% query coverage).

### 2.3. SCAR Marker Development and Examination

**Figure 4:**
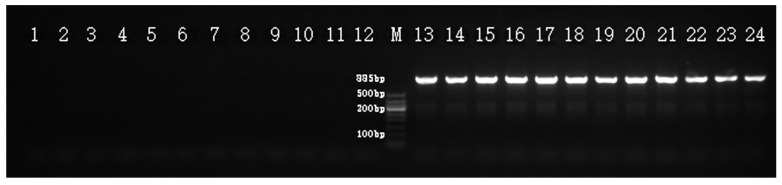
Amplification profile of the SCAR marker Hrcx-15 in *H. rhamnoides*, showing the 885 bp fragment, indicated with an arrow, in all female samples. Lanes 1–12: male, lanes 13–24: female. M, DNA Marker DL500 (Takara, Dalian, China).

According to the sequence of the RAPD-PCR products that were amplified with the D15 decamer primer, a converted SCAR marker (primer combination Hrcx-15-F and Hrcx-15-R) was produced that showed a single, distinct, and bright 885 bp band in all 140 pistillate genotypes, while no products were found in the 140 staminate genotypes (Figure 4). This 885 bp amplification product was named the Hrcx-15.

## 3. Discussion

Until now, reports about the genome sequence of *H. rhamnoides* were missing. RAPD analysis has the advantage to reveal a high degree of polymorphism without prior knowledge of the DNA sequence information of the species. However, to screen markers that are linked to sex determination genes on chromosomes via RAPD amplification depends on a variable success rate, because RAPD amplification is sensitive to the PCR reaction conditions with random decamer primers. In this context, we report a consistent and reproducible RAPD-PCR method for gender differentiation of *H. rhamnoides*.

Previously, various studies have explored sex-linked RAPD-SCAR markers in *H. rhamnoides*. According to Persson and Nybom [31], the approach of RAPD-PCR technology has been successfully applied to identify the sex of *H. rhamnoides*. Seventy-eight primers were tested and only one staminate-specific marker was qualified (OPD15-600). Out of the 31 decamer primers that were used by Satender et al. [32] to differentiate pistillate *H. rhamnoides* from staminate trees, only a single primer (OPF-11 with sequence 5'-TTGGTACCCC-3') produced an 1190-bp band, which resulted in a single gender-specific marker. This 1190-bp RAPD marker, which was consistently and reproducibly found in pistillate trees but was completely absent in male trees, can be used to select staminate and pistillate genotypes of *H. salicifolia*. A further attempt to recognize the gender-specific genes in *H. rhamnoides* was made by Sharma et al. [33], where the sex-linked RAPD marker OPD20-911 matched male plants. However, it is doubtful that the examination was confined to five staminate and pistillate genotypes only. Korekar et al. [34] ascertained two molecular SCAR markers linked to *H. rhamnoides*, by screening 60 RAPD primers from 20 male and 20 female bulk samples. Both female-specific markers (HrX1 and HrX2) were tested via a series of pistillate (120) and staminate (100) samples.

Forty-five RAPD primers were screened to identify a sex-linked marker among male and female genotypes of *H. rhamnoides* L. The random decamer primers exhibited variation in amplification patterns of the DNA samples, both within and between male and female genotypes. However, when these primers were tested on 140 individual male and female samples, only one primer, D15, amplified a female-specific fragment of approximately 0.9 kb consistently, whereas many primers generated putative sex-specific amplicons in some of the genotypes, but could not differentiate males from females reliably and stably. This situation is in sharp contrast with other studies on molecular markers linked to sex in other dioecious species. In *Pistacia vera* [35], about 400 primers were screened by bulked segregant analysis in order to find a 950-bp marker tightly linked to the female phenotype; in hop (*Humulus lupulus*), 1000 decamer primers were screened before the identification of a single marker useful for the construction of efficient SCAR discriminating male and female plants [36]. Finally, in asparagus (*Asparagus officinalis*), 760 primers were used in bulked segregant analysis in order to find a primer yielding two DNA fragments differentiating the two bulks, from which a SCAR marker associated with the M (male-determining) locus was developed [37]. We plan to screen many more random decamer primers to gain more molecular markers linked to the gender of *H. rhamnoides* L.

Based on the RAPD-PCR marker D15 that was linked to female *H. rhamnoides*, a pair of 20 oligonucleotide primers was obtained to apply highly specialized PCR amplification under stringent conditions. This guaranteed to reach a more convincing conclusion for distinguishing the early gender of *H. rhamnoides*. Gender identification of seedlings at the juvenile stage could help to revolutionize its future cultivation, as well as to investigate the sex determining mechanism of dioecious populations. The dioecious *H. rhamnoides* has an XX/XY sex chromosome. An X/Y chromosome sex determination system has already been described in a heteromorphic chromosome pair in staminate genotypes [38]. The total and relative length of the Y-chromosome surpasses the X-chromosome in size [20]. The development of a sex-linked nucleic acid sequence makes up a vast portion of the population, as shown in this paper, which suggests that the sex of *H. rhamnoides* is likely determined genetically.

RAPD primer-based PCR analysis has been commonly used to determine the sex of dioecious plants, but reproducibility of these primers exhibits its major disadvantage, because it is very sensitive to reaction conditions. However, SCAR markers are more independent to reaction conditions, and are usually dominant markers that can detect a single locus, making them more reproducible. The longer length (19–25 bp) and higher annealing temperatures of these primers make them more specific and stable. Because the SCAR marker Hrcx-15 was developed from female-specific RAPD fragments, the amplifications of the markers certainly indicate the femaleness of the samples, while no amplification directly shows the maleness of the sample. Nevertheless, the defect which could be imputed to the SCAR is that it mixes a non-amplification due to a failure in PCR with a male genotype, suah as DNA degradation. To solve this dilemma, a male-specific SCAR marker should be developed and used in a multiplex PCR with the female-specific SCAR marker Hrcx-15 in the future.

Additional markers need to be developed that are linked to gender-determination genes; this would allow the cloning of gene(s) involved in the process to generate SCAR markers. Despite the significant value of commercial plantation (allowing the differentiation of pistillate genotypes), these molecular markers would hereafter support the gene localization of x-linked locus in *H. rhamnoides*.

## 4. Materials and Methods

### 4.1. Plant Material

Green leaves of *H. rhamnoides* L. subsp. Sinensis Rousi were gathered in the Eastern region of Qinghai Province, China (Table 1). A total of 140 female and 140 male trees were discerned by a taxonomist during the fruiting month of August, when identification of staminate from pistillate genotypes was apparent. Green and tender leaves were plucked from both male and female trees and samples were kept at −80 °C for further research.

### 4.2. DNA Extraction

The genomic DNA of the green leaves of all trees was isolated via the Takara Plant Genomic DNA Extraction Kit (Takara, Dalian, China), after grinding plant tissue in liquid nitrogen. Both the concentration and quality of genomic DNA were measured via electrophoresis and an Epoch Microplate Spectrophotometer (BioTek Instruments, Winooski, VT, USA). Initially, two bulks, one from staminate genotypes and the other from pistillate genotypes, were mixed with equal mass of DNA from each of the 140 different-sexed plants.

### 4.3. RAPD Amplification

DNA amplifications of bulk DNA alleles from all 280 plants were screened with 45 random decamer primers for RAPD analysis (Sangon Biotech, Shanghai, China). The thermal cycler was programmed as reported by Williams et al. [39]. The final volume of 20 μL for PCR amplification reaction contained 1 U of Taq DNA polymerase (Takara, Dalian, China), 50 ng of isolated genomic DNA, Tris-HCl 10 mmol·L^−1^, MgCl_2_ 1.5 mmol·L^−1^, 200 μmol·L^−1^ each of dNTP, and random decamer primers 25 pmol·L^−1^. The PCR reactions were carried out in a Veriti^TM^ 96-well Thermal Cycler (Applied Biosystems, Life Technologies, Grand Island, New York, NY, USA) and amplified using the following conditions: 5 min of denaturation at 94 °C, a total of 45 1-min cycles at 94 °C, primer annealing for 1 min at 37 °C, a 2-min extension at 72 °C, and a final extension of 7 min at 72 °C. The amplification mixtures were refrigerated at 4 °C. PCR amplification products were run on 1.0% agarose gel via electrophoresis stained with 0.01% GelRed solution (Sangon Biotech, Shanghai, China) in 1 × TAE buffer. Gels were scanned via UV-light and photographs were taken via Azure cSeries imaging systems C150 analyzer (Azure Biosystems, Dublin, CA, USA). The size of DNA fragments was estimated via spotting Molecular DNA Marker DL2000 (Takara, Dalian, China).

### 4.4. Cloning of the RAPD Amplicon

The selective 885 bp polymorphism band was amplified by the decamer random primers D15, excised from 1.5% agarose gel, and was purified with an Agarose Gel DNA Extraction Kit (Takara, Dalian, China). The purified PCR products were mixed with an adenine tail using the sequencing vector pGEM^®^-T (Promega, Madison, WI, USA) using an optimum insert to vector ratio, and the mixtures were incubated at 4 °C overnight to obtain the maximum number of transformants. The ligation reaction was added into the JM109 high-efficiency competent with 45 s heat-shock in a 42 °C water bath [40]. The white positive colony was selected via blue/white color screening on indicator plates and the recombinant plasmid was obtained via overnight culture. Successful cloning of the insert was identified by amplifying the vector with the specific promoter primers SP6 and T7, which were also used for sequencing.

### 4.5. SCAR Marker Development and Examination

Sequences originated from the gender specific RAPD amplicon were used to design the SCAR primers via Primer Premier 6.0 software (PREMIER Biosof, Palo Alto, CA, USA). The primers Hrcx-15-F (5′-CATCCGTGCTTGCATAGAAT-3′) and Hrcx-15-R (5′-CATCCGTGCTCTCGGGAAGA-3′) for SCAR marker Hrcx-15 were designed and chemically synthesized. The PCR reaction was optimized with the following amplification conditions: in total, 30 cycles were performed, each cycle consisting of 30 s of initial denaturation at 94 °C, 30 s of annealing at 56 °C, and a 1-min extension at 72 °C. Before the start of the first cycle, amplifications were performed for 5 min at 94 °C, and a 7-min final extension at 72 °C took place after the final cycle. The amplification mixtures were investigated on a 1.5% agarose gel. The designed and synthesized SCAR primers were examined via genomic DNA from 140 staminate and 140 pistillate plants.

## 5. Conclusions

In conclusion, this study provides a female-specific SCAR marker Hrcx-15 for sex identification of *Hippophae rhamnoides* L. at very early developmental stages. This SCAR marker has proven to be a reproducible marker and can be applied by using PCR amplification as a quick and precise tool for discriminating females from male seedlings in *Hippophae rhamnoides* L. before transplantation into fields.

## Figures and Tables

**Table 1:** Information of the collected *H. rhamnoides* L. sample in the province of Qinghai.

Region	Elevation (m)	Longitude	Latitude
Mayigou reservoir, Huangzhong County	2644	E101°36′26.39′′	N36°29'34.76′′
Dahua Village, Huangyuan County	2745	E101°1058.77′′	N36°41′20.60′′
Chuchuer village, Pingan County	2875	E101°51′14.50′′	N36°23′33.24′′
Xiangyi village, Longhua County	2933	E102°02′40.21′′	N36°12′11.84′′
Tangfang village, Xunhua County	2544	E102°22′34.12′′	N35°49′41.65′′
Tiegeleng village, Xunhua County	2840	E102°37′27.01′′	N35°42′06.18′′
LangTang Village, Minhe County	2636	E102°44′10.64′′	N36°02′59.05′′
Xiaoshuiquan village, Pingan County	2556	E101°57′57.64′′	N36°29′29.05′′
Pandao village, Huangzhong County	2816	E101°21′03.40′′	N36°34′49.34′′
Youning temple, Minhe County	3007	E102°11′36.45′′	N36°45′10.16′′
Bianmagou Village, Datong County	3010	E101°50′40.05′′	N36°57′45.36′′
Double-tree village, Huzhu County	2436	E101°55′01.91′′	N36°46′47.96′′
Chengguan nursery, Datong County	2566	E101°33′12.67′′	N37°01′55.57′′
BaoKu Township, Datong County	2683	E101°33′59.65′′	N37°06′09.15′′
Botanical Garden, Xining City	2314	E101°44′38.09′′	N36°37'29.17′′
